# Revisiting the merits of a mandatory large group classroom learning format: an MD-MBA perspective

**DOI:** 10.1080/10872981.2017.1396174

**Published:** 2017-10-30

**Authors:** Shawn X. Li, Roshini Pinto-Powell

**Affiliations:** ^a^ Dartmouth Geisel School of Medicine, Hanover, NH, USA; ^b^ Dartmouth Tuck School of Business, Hanover, NH, USA

**Keywords:** MD-MBA, large group learning, mandatory class, network effect, competitive advantage

## Abstract

The role of classroom learning in medical education is rapidly changing. To promote active learning and reduce student stress, medical schools have adopted policies such as pass/fail curriculums and recorded lectures. These policies along with the rising importance of the USMLE (United States Medical Licensing Examination) exams have made asynchronous learning popular to the detriment of classroom learning. In contrast to this model, modern day business schools employ mandatory large group classes with assigned seating and cold-calling. Despite similar student demographics, medical and business schools have adopted vastly different approaches to the classroom.

When examining the classroom dynamic at business schools with mandatory classes, it is evident that there’s an abundance of engaging discourse and peer learning objectives that medical schools share. Mandatory classes leverage the network effect just like social media forums such as Facebook and Twitter. That is, the value of a classroom discussion increases when more students are present to participate. At a time when students are savvy consumers of knowledge, the classroom is competing against an explosion of study aids dedicated to USMLE preparation. Certainly, the purpose of medical school is not solely about the efficient transfer of knowledge – but to train authentic, competent, and complete physicians. To accomplish this, we must promote the inimitable and deeply personal interactions amongst faculty and students. When viewed through this lens, mandatory classes might just be a way for medical schools to leverage their competitive advantage in educating the complete physician.

## Framing the problem

Medical education is at a crossroads. While this sounds like an old familiar cry harkening back to the Flexner report 100 years ago, reviewing the recent literature on medical education, one observes changes in medical school curricula that have knowingly or unknowingly created a distinct change in the dynamic of learning [1]. The focus on perpetual knowledge acquisition, competition for awards and employment, and the ever-present specter of the USMLE exams (most notably USMLE Step 1), have created an unintended ‘culture of comparison’ [2]. Students have unwittingly acquired a mindset of competition and preparation for the board exams rather than collegiality and the purpose of knowledge acquisition for patient care.

Let us dissect one of the curricular changes that has become commonplace in most medical schools. Over the past three to five years, schools have seen a shift away from in-class attendance at lectures to students watching recorded lectures asynchronously. Many schools have moved completely away from large group lectures to small group learning (problem-based and case-based learning formats) and 89/142 LCME accredited schools in the US have moved to a Pass/Fail pre-clinical curriculum [3–5]. While each of these changes has well established merits, the sum total of these changes and the growing importance of performance on the Step 1 exam has contributed to a shift from learning for understanding to learning to perform well on the USMLE – an exam that most students believe will make or break their future career paths. A recent survey at our institution indicated that 39% of first and second-year students view the Step 1 exam as the most important aspect of their pre-clinical education (unpublished data). Educators and advisors at our institution and elsewhere are hard-pressed to tell students otherwise, as residency programs assign significant weight to the scores [6,7]. Given these trends, we feel that medical school educators will need to look carefully at the educational experiences we create to ensure that we are educating a workforce that can translate classroom learning into the desired patient care outcomes.

To put the discussion in context and create an educational parallel, at Dartmouth, the Geisel School of Medicine and the Tuck School of Business are located a stone’s throw from each other. Similar to Geisel, Tuck attracts a demographic of highly motivated and engaged students. Tuck’s stated educational mission is to ‘educate wise leaders to better the world of business’ and they are well known to have a robust and comprehensive general management program. In comparison to Geisel, at Tuck, classes are almost all mandatory and students have assigned seating with name cards. Students actively participate in classroom discussions and are also subject to ‘cold calling’ by the professor. Other peer business schools such as Harvard Business School and Haas School of Business at UC Berkley utilize similar classroom mechanics [8]. Observers who have visited and students who attend a Tuck class, can attest to the vibrant, engaging, as well as deeply informative nature of the large group mandatory classes. Students in attendance, are invited by cold calling to openly reason out loud and generate organic and thoughtful discussion regarding the topics being learned. Discussions are guided in a way to check prior understanding and knowledge and invite students to reflect on their rich and diverse experiences. On the other hand, medical schools (including ours) have been moving away from this large group format in the last decade, in pursuit of an ‘active’ and personalized curriculum. Mandatory large group sessions are being slowly phased out because they are thought to be a more passive form of learning. Efforts to give students more flexibility in the mode of learning is well intentioned, however, it is unclear whether these new curricula are an appropriate fit for the academic pressures and required skill building necessary to graduate competent physicians. As a consequence, while both medical and business schools share similar educational goals, they now pursue vastly different educational styles in the classroom. We believe it is time to ask medical educators, ‘Has the pendulum swung too far?’ Let us re-examine the merits of mandatory large group class in the context of learning in the 21st century.

## The classroom network

Practicing physicians recognize the team-based nature of modern medicine. To facilitate the progress of medicine and sustain life-long learning, doctors rely on themselves and their peers to collaborate on scientific research. Collaborations happen locally or across geographic regions for multi-center randomized control trials. Rarely do we push the envelope of medical knowledge in solitude. Likewise, in the practice of medicine, an interdisciplinary team is needed to deliver the highest quality care to our sickest patients. Hence, it is prudent that a medical school curriculum prepares students for the collaborative nature of being a physician. In other words, medical schools must engage students in the classroom setting so that students are not just learning, but learning *together*.

Learning together requires dialogue and discussion. To maximize the value of classroom discussions, we feel strongly that all students must be present in class. Classrooms are subject to network effects we see in social media forums such as Twitter and Facebook. That is, the value of a classroom discussion is dependent on how many students attend class. The more students who attend class, the more valuable the class is to each student [9]. Poorly attended lectures are uni-directional and little discussion is to be had. Students who experience this type of class may correctly conclude that watching the lecture at home will yield the same quality of learning. On the other hand, well attended and thoughtfully crafted lectures breed thought-provoking questions and vibrant discourse.

An active large group lecture is one of many methods developed to enhance interactive learning. In a systematic review by Fatmi et al. in 2013, a plurality of studies demonstrated that team-based learning was shown to be superior to alternatives for knowledge acquisition [10]. Furthermore, a 2015 scoping review on flipped classrooms, O’Flaherty and Phillips’ results indicate that although there was indirect evidence of improved academic performance and student and faculty satisfaction with the flipped classrooms, there was a paucity of conclusive evidence that the flipped classroom format contributed to building lifelong learning and skills [11]. A more recent 2017 systematic review on the effectiveness of flipped classrooms by Chen et al. confirm that while there were generally positive perceptions of the flipped classroom approach, the effect of flipped classrooms on changes in knowledge and skills were less conclusive in promoting knowledge acquisition above and beyond the more ‘traditional’ learning methods [12]. The review of evidence highlights the variations in teaching outcomes is probably dependent on many factors other than just the teaching format. Successful implementation of either the lecture or flipped classroom likely share a set of core competencies in active learning. Thus it is reasonable to postulate that regardless of the teaching format employed, the class must be engaging and interactive to be effective.

As demonstrated by the classes at Tuck, lectures and a large group teaching format can indeed actively and effectively involve the whole classroom to capitalize on the benefits demonstrated in team-based learning but also leverage the advantages of having all students participate in the discussion by drawing on the network effect. Pickering and Roberts described keys to the success of an active lecture which includes the relevance of content, space to check for understanding, and continued engagement and interaction [13]. Importantly, a large group of students offers a greater diversity of backgrounds from different socioeconomic strata that is reflective of a real-life community. After all, in medicine, learning often happens in the context of the real world.

For effective implementation of the active lecture, students not only have to be physically present, but mentally present as well. Cold calling is not to check whether students are on task, but to invite discussion for higher-order learning. Students are asked open-ended questions and encouraged to bring their previous experiences into the discussion. These methods are not meant to embarrass or pressure students, rather these methods of student engagement are meant to encourage all students to postulate, reason, and work through a problem out loud, without fear of being wrong. This is in contrast to small groups where dominant or quick thinking students can control the discussion, leaving those who do not process as quickly to feel left out of the discussion. Furthermore, research confirms that voluntary participation in discussions increases in classrooms with cold calling [14]. In medical education today, the pressure to appear smart and well-read is pushing learners of all stripes to be less explorative and creative in their thinking and to guard against the appearance of having knowledge gaps. Creating a safe learning environment and having robust faculty-facilitated discussions in mandatory classes can mitigate this alarming trend. We believe that if classroom sessions are worthy, students will attend and the ‘mandatory’ nature of classes will become moot.

## Competitive advantage of the classroom at a medical school

At the crux of this discussion about the benefits of required attendance is a more fundamental question – What is the purpose of classroom education? What is the value of having faculty and students in the same physical location for a defined period of time? The lecture style of teaching harkens back to a time when speaking to a large audience at the same time was the most efficient way to disseminate information. Today, there are many other forms of learning available to students that offer the same value proposition of efficiency and viewing students as informed consumers of knowledge is not outlandish. The focus on USMLE Step 1 and the explosive growth of study aids have created a situation whereby students are engaging in other forms of learning at the expense of classroom time [15,16]. If there is a medium of learning that satisfies the student’s needs better than the school’s curriculum, then they will choose it. Should medical schools compete with study aids solely on the dimension of learning efficiency? We suspect not. The competitive advantage of a medical school classroom environment is the rich and personal interaction between faculty experts and students. This interaction is impossible to replicate in any other forum. Coming full circle, the mandatory class can be seen as a method to capitalize on this competitive advantage. Medical schools are competing for the share of the student’s finite amount of time dedicated to the preparation of becoming a physician. There is more to learn about medicine than what is tested on board exams, and this is a competition that medical schools must win.

## Conclusion

The goal of a medical education has been, and always will be to educate authentic, competent, and skillful physicians of the future. Given the right circumstances, a well facilitated large group classroom session draws upon the network effect to elevate class discussions and to further both individual and group learning. The curriculum of the future could be a well-crafted hybrid within which students are prepared didactically in an engaging large group classroom format with fundamental concepts on which they elaborate in small group sessions. While we acknowledge that there are fundamental differences in the curriculum, goals, and process of educating MDs as compared to MBAs, we feel that much can be learned from our colleagues in business schools in understanding student needs, and delivering an engaging and valuable curriculum. Certainly, we are not suggesting that mandatory class is the solution for all the challenges in medical education today. However, by examining the merits of mandatory large group classroom sessions, we can begin to better understand a medical school’s competitive advantage in educating the complete physician. After all, it is imperative that in the profession of medicine, where the physician’s competency to communicate and reflect influences their ability to save a life, medical education ought to champion the same values.
